# PROFESSIONAL CAREGIVER SATISFACTION AND BURNOUT IN ASSISTED LIVING

**DOI:** 10.1093/geroni/igae098.3883

**Published:** 2024-12-31

**Authors:** Elizabeth Morton, Sheryl Zimmerman, Lea Efird-Green, Scott Davis

**Affiliations:** University of North Carolina at Chapel Hill, Chapel Hill, North Carolina, United States; University of North Carolina at Chapel Hill (UNC), Chapel Hill, North Carolina, United States; University of North Carolina at Chapel Hill, Chapel Hill, North Carolina, United States; University of North Carolina Eshelman School of Pharmacy, Asheville, North Carolina, United States

## Abstract

Professional caregivers (direct care workers) perform most of the care for residents in assisted living (AL) and have consistently high turnover rates. Because high turnover is associated with low job satisfaction and decreased quality of care, understanding professional caregiver satisfaction can inform approaches to improve the well-being of both staff and residents. This study obtained information from 559 professional caregivers from 69 AL communities to explore job satisfaction, burnout, and how satisfaction varies based on professional caregiver and AL characteristics (e.g., resident-case mix, staffing ratio). It also established the validity of the Direct Care Worker Job Satisfaction Scale (scored 1-4) as applied to this population through a principal components analysis. Principal components analysis produced a single-factor solution with an eigenvalue of 10.8, the only eigenvalue above 1. All scale items loaded at least 0.70 on the single factor. Overall, professional caregivers had an average satisfaction of 3.05 (“satisfied”), and 18.5% were burned out; satisfaction and burnout were negatively correlated (-0.46, p< 0.0001; adjusted -0.43, p< 0.0001). Satisfaction was positively correlated with the percent of residents with dementia (0.19, p< 0.0001) and provision of special/memory care at their AL community (0.17, p< 0.0001); it was negatively correlated with the professional caregiver-to-resident staffing ratio (-0.18, p< 0.0001) and presence of licensed nursing staff (-0.27, p< 0.0001). Results suggest that caring for individuals with dementia is particularly rewarding for professional caregivers, while being responsible for more residents and working alongside licensed nursing staff may lessen satisfaction.

